# The Mechanism of the Ordinal Position Effect: Stability Across Sense Modalities and the Hands Crossed Context

**DOI:** 10.1177/2041669519841071

**Published:** 2019-04-04

**Authors:** Qiangqiang Wang, Tingting Nie, Weixia Zhang, Wendian Shi

**Affiliations:** Department of Psychology, School of Education, Shanghai Normal University, China

**Keywords:** spatial-numerical association of response codes effect, ordinal position effect, mental number line, spatial representation, sense modality, automaticity, stability

## Abstract

The ordinal position effect posits that items positioned earlier in an ordinal sequence are responded to faster with the left key than the right key, and items positioned later in an ordinal sequence are responded to faster with the right key than the left key. Although the mechanism of the ordinal position effect has been investigated in many studies, it is unclear whether the ordinal position effect can extend to the auditory modality and the hands crossed context. Therefore, the present study employed days as the order information to investigate this question. Days were visually or acoustically displayed on a screen in random order, and participants were instructed to judge whether the probe day they perceived was before or after the current day (days-relevant task) or to identify the color or voice of the probe day they perceived (days-irrelevant task). The results indicate the following: (a) The days before the current day were responded to faster with the left key than the right key, and the days after the current day were responded to faster with the right key than the left key, both when the days-relevant task and the days-irrelevant task were performed, regardless of the sense modality. (b) The ordinal position effect for judgments of days was also obtained in the auditory modality even when the hands were crossed. These results indicate that the ordinal position effect can extend to the auditory modality and the hands crossed context, similar to the spatial-numerical association of response codes effect of numbers.

## Introduction

Dehaene and his collaborators displayed Arabic numerals ranging from one to nine (except five) one at a time centrally on a screen and then instructed participants to judge whether the probe number was an odd or even number by pressing the left or right key on a keyboard. They found that numbers smaller than five were responded to faster with left key presses than right. In contrast, numbers larger than five were responded to faster with right key presses, and the correlation of left-small number and right-large number was very strong. Dehaene, Bossini, and Giraux (1993) referred to this phenomenon as the spatial-numerical association of response codes (SNARC) effect. To explain this phenomenon, Dehaene et al. introduced the hypothesis of the mental number line (MNL) proposed by Restle, which postulates that numbers are spatially represented on the MNL by numerical magnitude from left to right. Specifically, small numbers are represented on the left of the MNL, and larger numbers are represented on the right of the MNL (Dehaene et al., 1993; Restle, 1970). Many subsequent researchers have found that the SNARC effect not only survives in the processing of Arabic numbers (Schwarz & Müller, 2006; Tan & Dixon, 2011; White, Szűcs, & Soltész, 2012; Wood, Willmes, Nuerk, & Fischer, 2008) but is also observed in the processing of other numerical and nonnumerical stimuli (e.g., pitch, luminance, and emotional pictures denoting different levels of happiness; Calabria & Rossetti, 2005; Cho, Bae, & Proctor, 2012; Fumarola et al., 2014; Holmes & Lourenco, 2011; Liu, Mai, & Fu, 2004; Kirjakovski & Utsuki, 2012). These studies further provided direct evidence for the MNL hypothesis. Thus, the SNARC effect is viewed as a gold standard to examine whether a particular stimulus is spatially represented.

Given that numbers simultaneously encompass the concept of quantity (how many) and serial order (which position; Jacob & Nieder, 2008), many researchers have employed the SNARC effect as an index for investigating whether order information is spatially represented in a manner similar to numbers (Casarotti, Michielin, Zorzi, & Umiltà, 2007; Dehaene et al., 1993; Dodd, Stigchel, Leghari, Fung, & Kingstone, 2008; Gevers, Reynvoet, & Fias, 2003, 2004; Ishihara, Keller, Rossetti, & Prinz, 2008). Although one study indicated that order information such as letters of the alphabet was not represented spatially because of the absence of the ordinal position effect in the processing of letters (Dehaene et al., 1993), most studies have indicated that order information was also spatially represented because the ordinal position effect was identified in the order information processing task (Abrahamse, van Dijck, Majerus, & Fias, 2014; Botvinick, & Watanabe, 2007; Gevers et al., 2003, 2004; Ginsburg, Archambeau, Van Dijck, Chetail, & Gevers, 2017; Huber, Klein, Moeller, & Willmes, 2016; Previtali, de Hevia, & Girelli, 2010; Zhang et al., 2016). For example, when Previtali et al. (2010) instructed participants to learn a list of nine words and then perform an order-relevant classification task or order-irrelevant classification task, the researchers were able to replicate the ordinal position effect in serial learning. Although most studies have found that all numbers and order information were represented spatially, several studies have indicated that the mechanism of the SNARC effect and the ordinal position effect was different (Casarotti et al., 2007; Dehaene et al., 1993; Dodd et al., 2008; Ginsburg & Gevers, 2015; Jan, Janosch, Telse, Marcus, & Sven, 2013; Nakhai, Pesciarelli, Mapelli, & Cacciari, 2012; Turconi, Campbell, & Seron, 2006; Wang, Liu, Shi, & Kang, 2018; Zhao et al., 2012). For example, Dodd et al. (2008) investigated whether letters, days, and months could shift individual attention in a way similar to numbers, and they found that while numbers could shift individual attention to the left or right according to numerical magnitude, the same was not true for letters, days, and months.

In addition, several previous studies investigated the stability of the SNARC effect across sense modalities, and the results have consistently indicated that the SNARC effect of numbers and numerical magnitudes occurred in both visual and auditory modalities (Golob, Lewald, Jungilligens, & Getzmann, 2016; Klein, Moeller, Nuerk, & Willmes, 2010; Kong, Zhao, & You, 2012; Nuerk, Wood, & Willmes, 2005). Moreover, accumulating evidence suggests that the SNARC effect of numbers is not exclusive to the hands uncrossed scenario, as was also observed when the participants crossed hands in the experiment (Dehaene et al., 1993; Viarouge, Hubbard, & Dehaene, 2014). Since the ordinal position effect was first identified in the processing of months and letters by Gevers (2003), most subsequent studies have further replicated the ordinal position effect in the processing of other order information (e.g., weeks, Chinese color words) and investigated the mechanism of this effect (Dodd et al., 2008; Gevers et al., 2004; Previtali et al., 2010; Zhang et al., 2016). Unfortunately, few studies have investigated whether the ordinal position effect in the processing of order information extends to the auditory modality passively or automatically, given the difference in the mechanism of the SNARC effect and the ordinal position effect. In addition, there was a difference in the SNARC effect between visual and auditory modalities, although this difference could not substantially inhibit the SNARC effect of numbers in the auditory modality (Szucs & Csépe, 2004; Zhao, Li, Deng, & Chen, 2017). Therefore, to discover the mechanism of the ordinal position effect, it is necessary to investigate whether the difference between visual and auditory modalities would inhibit the ordinal position effect of order information. Furthermore, no study has investigated whether, similar to the SNARC effect of numbers, the ordinal position effect is observed in the processing of order information when participants cross their hands in the experiment. Investigating these questions can also help to reveal the mechanism of the ordinal position effect. Thus, the present study aimed to investigate these subjects. Given that concepts such as the day before yesterday, yesterday, today, tomorrow, and the day after tomorrow are very familiar to everyone and not only encompass order information but also exclude magnitude information, we used this sequence of days as the order information to investigate the mechanism of the ordinal position effect in the present study. The study consisted of five experiments.

In the first two experiments, we visually presented days centrally on a Screen 1 at a time and instructed the participants either to judge whether the probe day was before today or after today (order-relevant task) or indicate the color of the probe day (order-irrelevant task). This information was collected to further consolidate and elaborate the ordinal position effect with new order information in order-relevant and order-irrelevant tasks. In addition, the results of these first two experiments were intended to provide a baseline comparison for the subsequent experiments of the present study. We hypothesized that the ordinal position effect would occur in each of the first two experiments.

Although previous studies have investigated the mechanism of the ordinal position effect, few studies have investigated whether the ordinal position effect could occur in the auditory modality. Therefore, in Experiment 3, this question was investigated by presenting the different days in the form of sound and instructing the participants to judge whether the days heard were before or after today.

Furthermore, nothing in the literature reports whether the ordinal position effect can be automatically activated in the auditory modality in the processing of order information. Thus, in Experiment 4, we investigated whether the ordinal position effect was automatically activated in the auditory modality when a days-irrelevant task was performed by presenting the different days using male and female voices and instructing the participants to judge whether the voices heard were male or female (days-irrelevant task).

Finally, several previous studies have indicated that the SNARC effect of numbers could not be inhibited when the hands were crossed (Dehaene et al., 1993; Viarouge et al., 2014). However, no study has investigated whether the ordinal position effect was impacted when the hands were crossed. Therefore, in Experiment 5, we investigated whether the ordinal position effect was influenced by the crossing of hands by asking the participants to cross their hands and perform the same classification task as in Experiment 4 of identifying whether the voice heard was male or female by pressing the left or right key.

## Experiment 1

### Methods

#### Participants

Thirty-eight right-handed students (22 females, 16 males, *M*_age_ = 22.55, *SD* = 2.14) from Shanghai Normal University voluntarily participated in this experiment. All participants had normal or corrected-to-normal vision and read or wrote from left to right. They received a payment of 5 Yuan RMB when the experiment ended. Informed consent forms were signed prior to starting the experiment. This experiment was approved by the ethics committee of the Shanghai Psychological Society (which also applies to the other experiments in the current study).

#### Stimuli and apparatus

The stimuli consisted of four words in black Chinese characters of similar stroke number and type (前天, 昨天, 明天, and 后天, which correspond to the day before yesterday, yesterday, tomorrow, and the day after tomorrow, respectively) printed in Times New Roman font (72 point size). All stimuli were presented on a 19″ computer screen (1024 × 768 resolution, refresh rate of 60 Hz).

#### Task and procedure

All experiments were conducted using E-prime1.0. When the experiment was initiated, a fixation first appeared in the center of the screen for 500 ms and was subsequently replaced by the probe stimulus. Once the probe stimulus appeared, the participants were instructed to judge whether the probe stimulus was before or after today and to enter their response by pressing one of the keys (left key corresponding to “F” and right key corresponding to “J”) with the left or right index finger as accurately and quickly as possible. The screen then displayed the participant’s response for 1500 ms before the next trial started. If the participant did not respond to the probe stimulus within 3 seconds, the trial was viewed as having an incorrect response, and the next trial was initiated after the display of a blank screen.

The entire experiment was composed of two blocks. In one block, the participants were instructed to press the left key with the left index finger in response to the days before today (yesterday and the day before yesterday) and the right key with their right index finger in response to the days after today (tomorrow and the day after tomorrow). In the other block, the relevant key to press was the opposite (i.e., the left key was pressed with the left index finger for the days after today and the right key with the right index finger for the days before today). The order of the blocks was balanced between the subjects. The entire experiment consisted of 88 trials (80 trials for the formal experiment and 8 trials for practice) and lasted approximately 10 minutes.

### Results and Discussion

There was no significant negative correlation between the reaction time and error rate across all trials, *r*(38) = −0.19, *p* > .05, which indicates that there was no speed–accuracy trade-off. Therefore, the error rate was not subjected to further analyses. The mean response times (RTs; all trials with incorrect responses and trials with RTs beyond three standard deviations were deleted under each treatment, for a total of 6.18% of trials excluded) were analyzed. A repeated measures analysis of variance (ANOVA) was conducted with day sequence (before today [yesterday, the day before yesterday] vs. after today [tomorrow, the day after tomorrow]) and side of response (left key vs. right key) as the within-subject factors.

**Figure 1.**
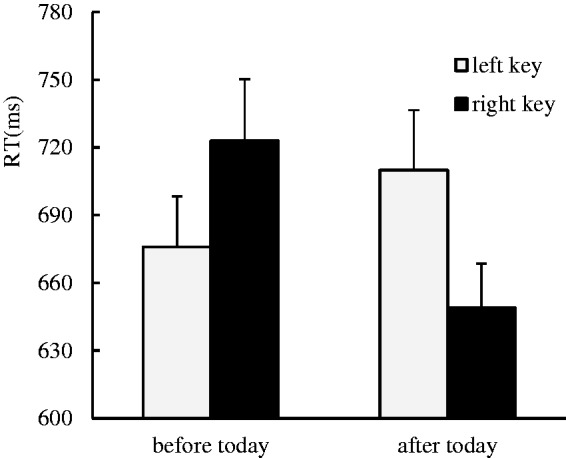
The ordinal position effect was identified in the days-relevant classification task, where the days before today were responded to faster with left key presses than right key presses, and the days after today were responded to faster with right key presses than left key presses. RT = response time.

The results indicated that there was no significant main effect for the side of response. There was a marginal significant main effect for the day sequence, *F*(1, 37) = 3.36, *p* = .075, η^2 ^= 0.083, in that the days after today (679 ± 20.91 ms) were responded to faster than the days before today (699 ± 22.69 ms), suggesting an advantage effect was captured in the processing of future time. A significant interaction effect between day sequence and side of response was identified, *F*(1, 37) = 8.95, *p* < .01, η^2 ^= 0.195. A further simple effect analysis showed that the days before today were responded to faster with left key presses (676 ± 22.41 ms) than right key presses (723 ± 27.26 ms), *F*(1, 37) = 5.21, *p* < .05, η^2 ^= 0.123, and the days after today were responded to faster with right key presses (649 ± 19.59 ms) than left key presses (710 ± 26.48 ms), *F*(1, 37) = 8.94, *p* < .01, η^2 ^= 0.195, which suggests that the ordinal position effect occurred in the processing of the day sequence (Figure 1).

In Experiment 1, we manipulated the day sequence to explore whether the ordinal position effect could be observed in the processing of the day sequence to further consolidate and elaborate the ordinal position effect. The results of our experiment were consistent with those of previous studies (Abrahamse et al., 2014; Gevers et al., 2003; Ginsburg et al., 2017; Huber et al., 2016; Previtali et al., 2010) and serve to consolidate and elaborate the ordinal position effect with new order information.

## Experiment 2

We replicated the ordinal position effect with the new order information in the days-relevant task in Experiment 1. Given the meaning of the word was automatically processed when an individual was forced to process the word’s color (Hoffmann, Hornung, Martin, & Schiltz, 2013; Stroop, 1935); in Experiment 2, we asked the participants to identify the color of the days presented to further consolidate and elaborate the automatic mechanism of the ordinal position effect in the visual modality with the days-irrelevant task.

### Methods

#### Participants

Thirty-eight right-handed university students (24 females, 14 males, *M*_age_ = 22.08, *SD* = 2.03) voluntarily participated in our experiment. As in Experiment 1, all participants exclusively read or wrote from left to right and had normal or corrected-to-normal vision. They received a payment of 5 Yuan RMB after the experiment ended. Informed consents were obtained prior to the start of the formal experiment.

#### Stimuli and apparatus

The stimuli were similar to those used in Experiment 1. The only difference lies in the colors of the stimuli. In the present experiment, the stimuli were either colored green or blank. The apparatus was identical to Experiment 1.

#### Task and procedure

We used the same procedure as in Experiment 1, except that the participants were instructed to judge whether the probe day was colored green or blank.

### Results and Discussion

Overall, a relatively low error rate was obtained (*M* = 2.01%, range: 0–11.25%). As no significant negative correlation between the RT and error rate across all trials was identified, *r*(38) = 0.06, *p* > .05, a speed-accuracy trade-off could be excluded. Accordingly, no further error rate analyses were necessary. The mean RTs (trials with incorrect responses and trials with RTs over three standard deviations in each condition were excluded, resulting in 3.91% of trials excluded) were subjected to further analyses. A repeated measures ANOVA was conducted with day sequence (before today [yesterday, the day before yesterday] vs. after today [tomorrow, the day after tomorrow]) and side of response (left key vs. right key) as the within-subject factors.

**Figure 2.**
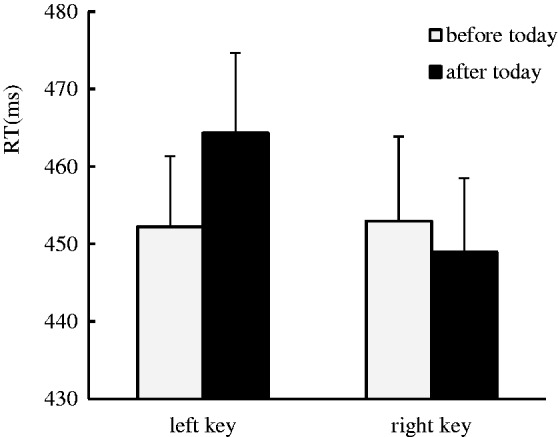
The ordinal position effect was identified in the visual modality when the days-irrelevant task was performed, where the days before today were responded to faster with left key presses than the days after today, and the days after today were responded to faster with right key presses than the days before today. RT = response time.

No significant main effect was identified for the side of response or the day sequence. Our data indicated a significant interaction effect between day sequence and side of response, *F*(1, 37) = 6.84, *p* < .05, η^2 ^= 0.156. The simple effect analysis showed that the days before today were responded to faster with left key presses (452 ± 9.09 ms) than the days after today (464 ± 10.34 ms), *F*(1, 37) = 5.02, *p* < .05, η^2 ^= 0.119, and the days after today were responded to slightly faster with right key presses (449 ± 9.55 ms) than the days before today (453 ± 10.92 ms), *F*(1, 37) = 0.77, *p* > .05, η^2 ^= 0.02, which suggests that the ordinal position effect occurred in the processing of the day sequence when the days-irrelevant task was performed (Figure 2).

In Experiment 2, the days-irrelevant task was used to explore whether the ordinal position effect of the day sequence captured in Experiment 1 was automatically activated to consolidate and elaborate the automatic mechanism of the ordinal position effect with the days-irrelevant task in the visual modality. Our results showed the ordinal position effect occurred in the processing of the day sequence when the days-irrelevant task was performed; therefore, from this result, we further confirm that the ordinal position effect can be automatically activated.

## Experiment 3

The previous two experiments consolidate and elaborate the ordinal position effect with new order information. Given that few studies have investigated whether the ordinal position effect could extend to the auditory modality, in Experiment 3, we further investigate this question.

### Methods

#### Participants

Thirty-four right-handed university students (22 females, 12 males, *M*_age_ = 22.65, *SD* = 1.87) voluntarily participated in our experiment. They all read or wrote from left to right and had normal or corrected-to-normal vision. All participants signed a written consent form prior to the start of the experiment and received a payment of 5 Yuan RMB after the experiment.

#### Stimuli and apparatus

The stimuli were auditory renditions of Chinese words (前天, 昨天, 明天, and 后天, which correspond to the day before yesterday, yesterday, tomorrow, and the day after tomorrow, respectively). Each Chinese word was read by a female voice in Mandarin Chinese and was presented via headphone. The audio format of these sound stimuli was PCM, the bit rate was 705 kbps, the audio sample size was 16 bit, and the sampling rate was 44100 Hz. Each sound stimulus lasted 450 ms. The apparatus was identical to that used in the preceding experiments.

#### Task and procedure

Except for the stimuli being delivered in an audio form, the task and procedure were identical to those in Experiment 1. The participants were instructed to judge whether the probe day heard was before or after today.

### Results and Discussion

A very low overall error rate was identified (*M* = 3.75%, range: 0–11.25%). There was no significant negative correlation between the reaction time and error rate across all trials, *r*(34) = 0.23, *p* > .05; thus, a speed-accuracy trade-off could be ruled out. Therefore, no further error rate analyses were conducted. The RTs (excluding trials with incorrect responses and trials with RTs over three standard deviations in each condition resulted in 5.96% of trials being excluded) were further analyzed. A repeated measures ANOVA was conducted with day sequence (before today [yesterday, the day before yesterday] vs. after today [tomorrow, the day after tomorrow]) and side of response (left key vs. right key) as the within-subject factors.

**Figure 3.**
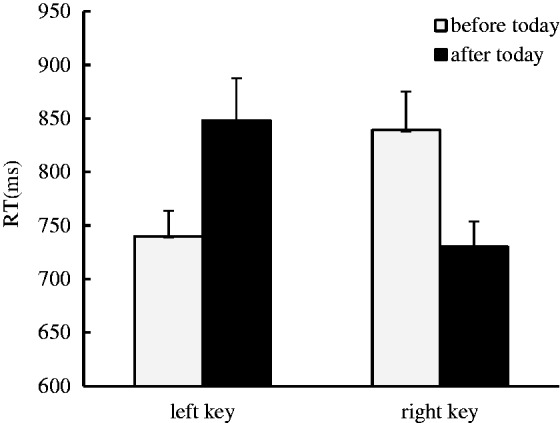
The ordinal position effect identified in the auditory modality when the days-relevant task was performed, where the days before today were responded to faster with left key presses than the days after today, and the days after today were responded to faster with right key presses than the days before today. RT = response time.

No significant main effect was identified. Interestingly, a significant interaction effect between the day sequence and the side of response was identified, *F*(1, 33) = 16.29, *p* < .001, η^2 ^= 0.33. The simple effect analysis showed that the days before today were responded to faster with left key presses (739 ± 23.53 ms) than the days after today (847 ± 39.58 ms), *F*(1, 33) = 13.22, *p* = .001, η^2 ^= 0.286, and the days after today were responded to faster with right key presses (730 ± 23.72 ms) than the days before today (839 ± 36.07 ms), *F*(1, 33) = 13.07, *p* = .001, η^2 ^= 0.284, which suggests that the ordinal position effect of day sequence occurred in the auditory modality when the days-relevant task was performed (Figure 3).

In Experiment 3, the days were presented in an auditory form, and the participants were instructed to judge whether the probe day was before or after today. The ordinal position effect was identified in this experiment; therefore, we conclude that the ordinal position effect of order information can extend to the auditory modality when an order-relevant task is performed.

## Experiment 4

In Experiment 3, we found that the ordinal position effect could extend to the auditory modality, but we still could not be sure whether the ordinal position effect would be automatically captured in the auditory modality in the order-irrelevant task. Therefore, Experiment 4 aimed to further investigate whether the ordinal position effect was automatically activated in the auditory modality.

### Methods

#### Participants

Thirty-two right-handed university students (25 females, 7 males, *M*_age_ = 22.69, *SD* = 1.52) voluntarily participated in our experiment. All participants had normal or corrected-to-normal vision and read or wrote from left to right. A written consent form was obtained from all participants prior to the experiment. They received a payment of 5 Yuan RMB when the experiment ended.

#### Stimuli and apparatus

With respect to the stimuli and apparatus, the only difference between Experiments 3 and 4 lies in the form in which the sound stimulus was presented. In Experiment 4, all sound stimuli were in Mandarin Chinese spoken by a female or male voice. All other parameters of the sound stimuli were the same as those used in Experiment 3.

#### Task and procedure

Other than the form of stimulus delivery, no difference existed between Experiments 1 and 4. The days were presented in an auditory form in Experiment 4, and the participants were instructed to judge whether the probe day heard was read in a male or female voice.

### Results and Discussion

We found a very low overall error rate in the experiment (*M* = 2.81%, range: 0–10%). In addition, because there was no significant negative correlation between the RT and error rate across all trials, *r*(32) = 0.12, *p* > .05, a speed-accuracy trade-off could be ruled out. Therefore, no further error rate analyses were conducted. The mean RTs (after excluding trials with incorrect responses and trials with RTs beyond three standard deviations in each condition, 95.08% of trials remained) were subjected to further analyses. A repeated measures ANOVA was conducted with day sequence (before today [yesterday, the day before yesterday] vs. after today [tomorrow, the day after tomorrow]) and side of response (left key vs. right key) as the within-subject factors.

**Figure 4.**
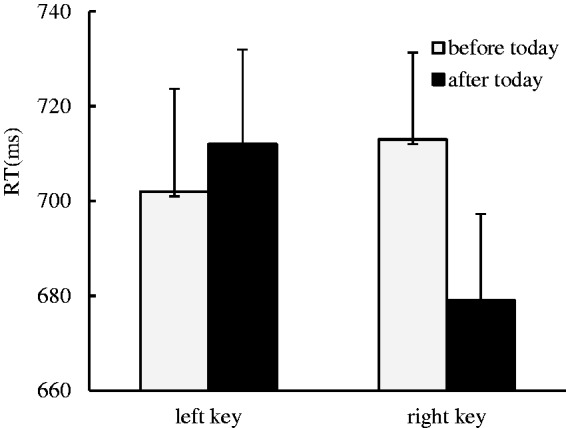
The ordinal position effect of the day sequence was identified in the auditory modality when the days-irrelevant task was performed, where the days before today were responded to faster with left key presses than the days after today, and the days after today were responded to faster with right key presses than the days before today. RT = response time.

Our data indicated that there was no significant main effect for the side of response. There was a marginal significant main effect for the day sequence, *F*(1, 31) = 3.48, *p* = .071, η^2 ^= 0.101, where the days after today (696 ± 18.58 ms) were responded to much faster than the days before today (707 ± 19.12 ms), suggesting an advantage effect was captured in the processing of future time. We identified a significant interaction effect between the day sequence and the side of response, *F*(1, 31) = 12.50, *p* = .001, η^2 ^= 0.287. A further simple effect analysis showed that the days before today were responded to slightly faster with left key presses (702 ± 21.64 ms) than the days after today (712 ± 19.98 ms), *F*(1, 31) = 1.49, *p* > .05, η^2 ^= 0.046, and the days after today were responded to faster with right key presses (679 ± 18.24 ms) than the days before today (713 ± 18.33 ms), *F*(1, 31) = 13.64, *p* = .001, η^2 ^= 0.305, which suggests that the ordinal position effect occurred in the auditory modality when the days-irrelevant task was performed (Figure 4).

In Experiment 4, to investigate whether the ordinal position effect was automatically activated, the sound stimuli were presented via a female or male voice, and the participants were instructed to judge whether the voice they heard was female or male. The results indicated an ordinal position effect. Thus, we conclude that the ordinal position effect was automatically activated in the auditory modality.

## Experiment 5

Through Experiments 3 and 4, we confirmed that the ordinal position effect could be extended to the auditory modality, regardless of what task is performed. In Experiment 5, we further investigate whether the ordinal position effect can extend to the hands crossed context.

### Methods

#### Participants

Thirty-four right-handed university students (24 females, 10 males, *M*_age_ = 21.03, *SD* = 3.27) voluntarily participated in our experiment. All participants had normal or corrected-to-normal vision and read or wrote from left to right. A written consent form was obtained from each participant prior to the experiment. Each participant received a payment of 5 Yuan RMB when the experiment ended.

#### Stimuli and apparatus

The stimuli and apparatus were the same as those used in Experiment 4, except that the participants crossed their hands in Experiment 5.

#### Task and procedure

Other than the position of the hands, the task and procedure were similar to Experiment 4. Specifically, in Experiment 5, the participants were asked to press the left key with the right index finger and press the right key with the left index finger to indicate whether the probe day heard was read by a male or female voice.

### Results and Discussion

We found a very low overall error rate in the experiment (*M* = 3.97%, range: 0–8.75%). In addition, because there was no significant positive correlation between the RT and error rate across all trials, *r*(34) = 0.203, *p* > .05, a speed-accuracy trade-off could be ruled out. Therefore, no further error rate analyses were conducted. The mean RTs (after excluding trials with incorrect responses and trials with RTs beyond three standard deviations in each condition, 94.24% of trials remained) were subjected to further analyses. A repeated measures ANOVA was conducted with day sequence (before today [yesterday, the day before yesterday] vs. after today [tomorrow, the day after tomorrow]) and side of response (left key vs. right key) as the within-subject factors.

**Figure 5.**
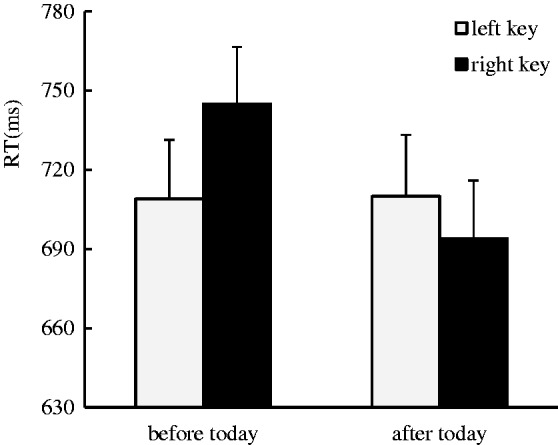
The ordinal position effect was automatically activated in the auditory modality when the hands were crossed, where the days before today were responded to faster with left key presses than right key presses, and the days after today were responded to faster with right key presses than left key presses. RT = response time.

Our data indicated that there was no significant main effect for the side of response. There was a significant main effect for the day sequence, *F*(1, 33) = 14.66, *p* = .001, η^2 ^= 0.308, in which the days after today (702 ± 22.25 ms) were responded to much faster than the days before today (727 ± 21.31 ms), suggesting an advantage effect was captured in the processing of future time. We identified a significant interaction effect between the day sequence and the side of response, *F*(1, 33) = 17.10, *p* < .001, η^2 ^= 0.287. A further simple effect analysis showed that the days before today were responded to faster with left key presses (709 ± 22.34 ms) than right key presses (745 ± 21.58 ms), *F*(1, 33) = 11.21, *p* < .01, η^2 ^= 0.254, and the days after today were responded to slightly faster with right key presses (694 ± 21.96 ms) than the days before today (710 ± 23.32 ms), *F*(1, 33) = 3.41, *p* = .074, η^2 ^= 0.094, which suggests that the ordinal position effect automatically occurred in the auditory modality when the hands were crossed (Figure 5).

In Experiment 5, to investigate whether the ordinal position effect occurred in the hands crossed context, the sound stimuli were presented via a female or male voice, and the participants were instructed to cross their hands and then judge whether the probe stimuli were read by a female or male voice. The results indicated an ordinal position effect as well. Thus, we concluded that the ordinal position effect can be observed when the hands are crossed.

## General Discussion

Previous studies have indicated that the SNARC effect generally occurred in the visual modality regardless of the classification task performed. The research also found that this effect could extend to the auditory modality and the hands crossed context. Although several studies have indicated that the ordinal position effect widely occurred in the processing of order information in the visual modality when both order-relevant and order-irrelevant tasks were performed, little was known about whether the ordinal position effect could extend to the auditory modality and could be observed when the hands were crossed, similar to the SNARC effect of numbers. Therefore, the present study used days, such as the day before yesterday, yesterday, today, tomorrow, and the day after tomorrow, as new order information to investigate these questions.

In the first two experiments, we aimed to further consolidate and elaborate the ordinal position effect with new order information using order-relevant and order-irrelevant tasks in the visual modality. The results indicated that the ordinal position effect can be observed in the processing of the days used in the present study. These results were consistent with many previous studies that have suggested that the ordinal position effect could occur in the processing of order information both in the performance of order-relevant and order-irrelevant tasks (Gevers et al., 2004; Ginsburg & Gevers, 2015; Previtali et al., 2010; Prpic et al., 2016; van Dijck, Abrahamse, Majerus, & Fias, 2013; van Dijck & Fias, 2011; Zhang et al., 2016). The result from the first two experiments thus indicated that it was feasible to investigate the mechanism of the ordinal position effect using a sequence of days such as the day before yesterday, yesterday, today, tomorrow, and the day after tomorrow. Therefore, in the subsequent experiments of the present study, we employed this same sequence of days as the order information to further investigate the mechanism of the ordinal position effect in the auditory modality and the hands crossed context. Although we replicated the ordinal position effect with this day sequence, our results still differed from those of previous studies. For example, Gevers et al. used the days of the week as order information to investigate whether the ordinal position effect occurred in the processing of ordinal information. In their study, participants were required to indicate whether a given day came before or after Wednesday and the results showed faster responses with the left hand for Monday–Tuesday and, conversely, faster right-hand responses for Thursday and Friday. However, in the data of Gevers, the positive difference in RT between the right and left hand for the days before Wednesday was small indeed (approximately 5–10 ms), while the negative difference for the days after Wednesday was slightly larger but still approximately 20 ms (Gevers et al., 2004). Notably, in the present study, the difference in reaction time between right and left hand for the days before today (and vice versa for those after today) is much greater, approximately 50 to 60 ms. What accounts for this difference? It is likely the result of the different frames of reference used by the two studies (Wednesday vs. today). In the study of Gevers, Wednesday was used as the frame of reference, and participants were asked to classify whether the probe day was before or after Wednesday, but in the present study, the frame of reference was today, and participants were asked to classify whether the probe day was before or after today. Day designations such as the day before yesterday, yesterday, today, tomorrow, and the day after tomorrow are well known to children before they are enrolled in school. However, children often start learning the days of the week only after they are enrolled in school. In addition, although weeks are familiar to students because they are often used to define students’ course schedules and holidays, individuals often use today as a reference frame, rather than a particular day of the week, to look back at the past and look forward to the future. For example, an individual often can remember what they did yesterday on the first try, but they usually fail to remember what they did on which day of the week on the first try. Sometimes, individuals do not even know which day of the week today is. Therefore, days of the week are less familiar to students compared with days such as the day before yesterday, yesterday, today, tomorrow, and the day after tomorrow. Familiarity can promote automaticity in cognitive processes (Toth, 1996). Thus, using today as a reference frame can more easily and quickly activate this association between space and the probe day compared with using Wednesday as the reference frame.

Only a few studies have investigated whether the ordinal position effect could extend to the auditory modality, although the SNARC effect of numbers has been shown to occur even in the auditory modality (Golob, et al., 2016; Nuerk et al., 2005). After consolidating and elaborating the ordinal position effect by using new order information in the form of a sequence of days (namely, the day before yesterday, yesterday, today, tomorrow, the day after tomorrow) in the first two experiments, we further investigated whether the ordinal position effect could extend to the auditory modality, similar to the SNARC effect of numbers. In Experiment 3, the days were randomly presented in the form of sound, and participants were instructed to judge whether the probe day they heard was before or after today. The ordinal position effect was identified in Experiment 3. Therefore, we concluded that the ordinal position effect could extend to the auditory modality, similar to the SNARC effect of numbers and numerical magnitudes. To further investigate whether the ordinal position effect in the auditory modality could be automatically activated, in Experiment 4, the days were presented via a female or male voice, and participants were instructed to judge whether the probe day was read by a female or male (order-irrelevant task). The ordinal position effect was observed in Experiment 4 as well. Based on this result, it could be concluded that the ordinal position effect could also be automatically activated in the auditory modality. Previous studies have investigated the mechanism of the SNARC effect of numbers in the auditory modality. Few studies have investigated the mechanism of the ordinal position effect in the auditory modality. Therefore, the present study investigated the mechanism of the ordinal position effect in Experiments 3 and 4. The results of these experiments indicated that the ordinal position effect could occur in the auditory modality and could be automatically activated, similar to the SNARC effect of numbers. The results of these experiments further supported previous studies by expanding the modalities affected by the order position effect and further enriched the research on the ordinal position effect in this field.

In addition to investigating whether the ordinal position effect could extend to the auditory modality, in Experiment 5, we further investigated whether the ordinal position effect could occur when the hands were crossed, similar to the SNARC effect of numbers. In Experiment 5, the days were presented via a male or female voice, and participants were instructed to cross their hands and judge whether the probe day heard was read by a male or female voice by pressing the left or right key as in Experiment 4. The results showed that the ordinal position effect also occurred in the hands crossed context, suggesting that the ordinal position effect could not be influenced by the position of the hands, similar to the SNARC effect of numbers. From this result, we can conclude that the ordinal position effect is likewise independent of effectors.

Although many previous studies have suggested that the mechanism behind the SNARC effect and the ordinal position effect may be different (Ginsburg & Gevers, 2015; Prpic et al., 2016; Turconi et al., 2006; Zhao et al., 2012), Gevers et al. nevertheless have indicated that a (cor)relation probably exists between processing numbers and processing order information (Gevers et al., 2004). This position was supported by a recent imaging study in which Fias, Lammertyn, Caessens, and Orban (2007). showed that the same cortical network was activated in the processing of numbers and nonnumerical order information. Most previous studies have indicated that the SNARC effect of numbers could extend to the auditory modality and that it also occurred when the hands were crossed (Dehaene et al., 1993; Golob et al., 2016; Klein et al., 2010; Kong et al., 2012; Nuerk et al., 2005; Viarouge et al., 2014). The present study further investigated whether the ordinal position effect could also extend to the auditory modality and the hands crossed context. The results showed that the ordinal position effect occurred both in the auditory modality and the hands crossed context. The present results provide further support for the position that a (cor)relation probably exists between the processing of numbers and the processing of order information.

## Conclusion

From the present study, we conclude the following: (a) The ordinal position effect can extend to the auditory modality and the hands crossed context. (b) The ordinal position effect can be automatically activated, regardless of the sense modality.

## References

[bibr1-2041669519841071] Abrahamse E. van Dijck J. P. Majerus S. Fias W. (2014) Finding the answer in space: The mental whiteboard hypothesis on serial order in working memory. Frontiers in Human Neuroscience 8: 1–10. 25505394 10.3389/fnhum.2014.00932 PMC4243569

[bibr2-2041669519841071] Botvinick M. Watanabe T. (2007) From numerosity to ordinal rank: A gain-field model of serial order representation in cortical working memory. Journal of Neuroscience the Official Journal of the Society for Neuroscience 27: 8636–8642. 10.1523/JNEUROSCI.2110-07.2007 PMC6672950 17687041

[bibr3-2041669519841071] Calabria M. Rossetti Y. (2005) Interference between number processing and line bisection: A methodology. Neuropsychologia 43: 779–783. 15721190 10.1016/j.neuropsychologia.2004.06.027

[bibr4-2041669519841071] Casarotti M. Michielin M. Zorzi M. Umiltà C. (2007) Temporal order judgment reveals how number magnitude affects visuospatial attention. Cognition 102: 101–117. 17046735 10.1016/j.cognition.2006.09.001

[bibr5-2041669519841071] Cho Y. S. Bae G. Y. Proctor R. W. (2012) Referential coding contributes to the horizontal SMARC effect. Journal of Experimental Psychology Human Perception & Performance 38: 726–734. 22141586 10.1037/a0026157

[bibr6-2041669519841071] Dehaene S. Bossini S. Giraux P. (1993) The mental representation of parity and number magnitude. Journal of Experimental Psychology General 122: 371–396.

[bibr7-2041669519841071] Dodd M. D. Stigchel S. V. D. Leghari M. A. Fung G. Kingstone A. (2008) Attentional SNARC: There’s something special about numbers (let us count the ways). Cognition 108: 810–818. 18538756 10.1016/j.cognition.2008.04.006

[bibr8-2041669519841071] Fias W. Lammertyn J. Caessens B. Orban G. A. (2007) Processing of abstract ordinal knowledge in the horizontal segment of the intraparietal sulcus. Journal of Neuroscience 27: 8952–8956. 17699676 10.1523/JNEUROSCI.2076-07.2007 PMC6672167

[bibr9-2041669519841071] Fumarola A. Prpic V. Pos O. D. Murgia M. Umiltà C. Agostini T. (2014) Automatic spatial association for luminance. Attention Perception & Psychophysics 76: 759–765. 10.3758/s13414-013-0614-y 24402699

[bibr10-2041669519841071] Gevers W. Reynvoet B. Fias W. (2003) The mental representation of ordinal sequences is spatially organized. Cognition 87: B87–B95. 12684205 10.1016/s0010-0277(02)00234-2

[bibr11-2041669519841071] Gevers W. Reynvoet B. Fias W. (2004) The mental representation of ordinal sequences is spatially organized: Evidence from days of the week. Cortex 40: 171–172. 15174454 10.1016/s0010-9452(08)70938-9

[bibr12-2041669519841071] Ginsburg V. Archambeau K. Van Dijck J. P. Chetail F. Gevers W. (2017) Coding of serial order in verbal, visual and spatial working memory. Journal of Experimental Psychology General 146: 632–650. 28459262 10.1037/xge0000278

[bibr13-2041669519841071] Ginsburg V. Gevers W. (2015) Spatial coding of ordinal information in short- and long-term memory. Frontiers in Human Neuroscience 9: 1–10. 25688199 10.3389/fnhum.2015.00008 PMC4311612

[bibr14-2041669519841071] Golob E. J. Lewald J. Jungilligens J. Getzmann S. (2016) Interaction of number magnitude and auditory localization. Perception 45: 165–179. 26562857 10.1177/0301006615599906

[bibr15-2041669519841071] Hoffmann D. Hornung C. Martin R. Schiltz C. (2013) Developing number-space associations: SNARC effects using a color discrimination task in 5-year-olds. Journal of Experimental Child Psychology 116: 775–791. 24055929 10.1016/j.jecp.2013.07.013

[bibr16-2041669519841071] Holmes K. J. Lourenco S. F. (2011) Common spatial organization of number and emotional expression: A mental magnitude line. Brain & Cognition 77: 315–323. 21839568 10.1016/j.bandc.2011.07.002

[bibr17-2041669519841071] Huber S. Klein E. Moeller K. Willmes K. (2016) Spatial-numerical and ordinal positional associations coexist in parallel. Frontiers in Psychology 7: 1–13. 27064216 10.3389/fpsyg.2016.00438 PMC4811880

[bibr18-2041669519841071] Ishihara M. Keller P. E. Rossetti Y. Prinz W. (2008) Horizontal spatial representations of time: Evidence for the STEARC effect. Cortex 44: 454–461. 18387578 10.1016/j.cortex.2007.08.010

[bibr19-2041669519841071] Jacob S. N. Nieder A. (2008) The ABC of cardinal and ordinal number representations. Trends in Cognitive Sciences 12: 0–43. 10.1016/j.tics.2007.11.006 18178515

[bibr20-2041669519841071] Jan L. Janosch L. Telse N. Marcus H. Sven L. (2013) Spatial representations of numbers and letters in children. Frontiers in Psychology 4: 1–5. 23970877 10.3389/fpsyg.2013.00544 PMC3748714

[bibr21-2041669519841071] Kirjakovski A. Utsuki N. (2012) From SNARC to SQUARC: Universal mental quantity line? International Journal of Psychological Studies 4: 217–227.

[bibr22-2041669519841071] Klein E. Moeller K. Nuerk H. C. Willmes K. (2010) On the neuro-cognitive foundations of basic auditory number processing: An FMRI study. Behavioral & Brain Functions 6: 1–13. 20618926 10.1186/1744-9081-6-42 PMC2911396

[bibr23-2041669519841071] Kong F. Zhao J. You X. (2012) Components representation of negative numbers: Evidence from auditory stimuli detection and number classification tasks. Quarterly Journal of Experimental Psychology 65: 691–701. 10.1080/17470218.2011.622048 22133233

[bibr24-2041669519841071] Liu C. Mai X. Fu X. (2004) The spatial numerical association of response codes effect of number processing in different attention conditions. Acta Psychologica Sinica 36: 671–680.

[bibr25-2041669519841071] Nakhai S. Pesciarelli F. Mapelli D. Cacciari C. (2012) Can perceiving letters cause spatial shifts of attention? Procedia—Social and Behavioral Sciences 32: 79–81.

[bibr26-2041669519841071] Nuerk H. C. Wood G. Willmes K. (2005) The universal SNARC effect. Experimental Psychology 52: 187–194. 16076066 10.1027/1618-3169.52.3.187

[bibr27-2041669519841071] Previtali P. de Hevia M. D. Girelli L. (2010) Placing order in space: The SNARC effect in serial learning. Experimental Brain Research 201: 599–605. 19888566 10.1007/s00221-009-2063-3

[bibr28-2041669519841071] Prpic V. Fumarola A. De Tommaso M. Luccio R. Murgia M. Agostini T. (2016) Separate mechanisms for magnitude and order processing in the spatial-numerical association of response codes (SNARC) effect: The strange case of musical note values. Journal of Experimental Psychology Human Perception & Performance 42: 1241–1251. 26950384 10.1037/xhp0000217

[bibr29-2041669519841071] Restle F. (1970) Speed of adding and comparing numbers. Journal of Experimental Psychology 83: 274–278.

[bibr30-2041669519841071] Schwarz W. Müller D. (2006) Spatial associations in number-related tasks: A comparison of manual and pedal responses. Experimental Psychology 53: 4–15. 16610269 10.1027/1618-3169.53.1.4

[bibr31-2041669519841071] Stroop J. R. (1935) Studies of interference in serial verbal reactions. Journal of Experimental Psychology General 121: 15–23.

[bibr32-2041669519841071] Szucs D. Csépe V. (2004) Access to numerical information is dependent on the modality of stimulus presentation in mental addition: A combined ERP and behavioral study. Cognitive Brain Research 19: 10–27. 14972354 10.1016/j.cogbrainres.2003.11.002

[bibr33-2041669519841071] Tan S. Dixon P. (2011) Repetition and the SNARC effect with one- and two-digit numbers. Canadian Journal of Experimental Psychology 65: 84–97. 21668090 10.1037/a0022368

[bibr34-2041669519841071] Turconi E. Campbell J. I. Seron X. (2006) Numerical order and quantity processing in number comparison. Cognition 98: 273–285. 16399265 10.1016/j.cognition.2004.12.002

[bibr35-2041669519841071] Toth J. P. (1996) Conceptual automaticity in recognition memory: Levels-of-processing effects on familiarity. Canadian Journal of Experimental Psychology 50: 123–138. 8653094 10.1037/1196-1961.50.1.123

[bibr36-2041669519841071] van Dijck J. P. Abrahamse E. L. Majerus S. Fias W. (2013) Spatial attention interacts with serial-order retrieval from verbal working memory. Psychological Science 24: 1854–1859. 23863755 10.1177/0956797613479610

[bibr37-2041669519841071] van Dijck J. P. Fias W. (2011) A working memory account for spatial-numerical associations. Cognition 119: 114–119. 21262509 10.1016/j.cognition.2010.12.013

[bibr38-2041669519841071] Viarouge A. Hubbard E. M. Dehaene S. (2014) The organization of spatial reference frames involved in the SNARC effect. The Quarterly Journal of Experimental Psychology 67: 1484–1499. 24571534 10.1080/17470218.2014.897358

[bibr39-2041669519841071] Wang Q. Liu M. Shi W. Kang J. (2018) Mechanism of the SNARC effect in numerical magnitude, time sequence, and spatial sequence tasks: Involvement of LTM and WM. Frontiers in Psychology 9: 1–12. 30186215 10.3389/fpsyg.2018.01558 PMC6110948

[bibr40-2041669519841071] White S. L. J. Szűcs D. Soltész F. (2012) Symbolic number: The integration of magnitude and spatial representations in children aged 6 to 8 years. Frontiers in Psychology 2: 392. 22291671 10.3389/fpsyg.2011.00392 PMC3249610

[bibr41-2041669519841071] Wood G. Willmes K. Nuerk H. C. Fischer M. H. (2008) On the cognitive link between space and number: A meta-analysis of the SNARC effect. Psychology Science 50: 489–525.

[bibr42-2041669519841071] Zhang M. Gao X. Li B. Yu S. Gong T. Jiang T. Chen Y. (2016) Spatial representation of ordinal information. Frontiers in Psychology 7: 1–6. 27092100 10.3389/fpsyg.2016.00505 PMC4823274

[bibr43-2041669519841071] Zhao H. Chen C. Zhang H. Zhou X. Mei L. Chen C. Dong Q. (2012) Is order the defining feature of magnitude representation? an ERP study on learning numerical magnitude and spatial order of artificial symbols. Plos One 7: 1–9. 10.1371/journal.pone.0049565 PMC3501518 23185363

[bibr44-2041669519841071] Zhao Q. Li F. Deng Z. Chen Y. (2017) Characteristics of the spatial representation of magnitude: SNARC effect across audiovisual channels. Psychological Development and Education 33: 129–138.

